# Through the looking glass: a review of the literature surrounding reflective practice in dentistry

**DOI:** 10.1038/s41415-022-3993-4

**Published:** 2022-05-27

**Authors:** Faith Campbell, Helen Rogers

**Affiliations:** 41415106785001 Glasgow Dental Hospital and School; Glasgow, UK; Honorary Clinical Teacher in Paediatric Dentistry, Academic Unit of Oral Health, Dentistry and Society, School of Clinical Dentistry, University of Sheffield, UK; 41415106785002 School of Dental Sciences, Newcastle University, UK

## Abstract

Reflection is an essential component of the learning process that helps to elicit deeper learning. In healthcare, this uses experiential activities to produce knowledge that compels the clinician to change their practice. Deep reflection allows one to explore emotions associated with challenging learning experiences, empowering reinterpretation of these experiences and removing barriers to further learning. Reflection is a key requirement of dental education at all stages. This paper aims to explore the existing literature on reflective practice in dentistry and identify areas for further research to improve reflective practice within dentistry.

Traditional methods of reflecting through written means are not facilitating the deep reflection which is desired. A systematic overhaul of reflective practice is suggested, involving a shift away from structured written reflections. There is little evidence to inform the most appropriate format for reflective practice in dental education. There is a need for further research to determine the effectiveness of reflective practice in dental education, particularly as a move away from structured written reflection to more creative reflective opportunities are encouraged. Greater exploration of barriers to reflection in dentistry is indicated, with consideration to how these may be overcome and a need to engage regulatory bodies in system-wide changes.

## Key points

- Reflection is an essential aspect of the learning process, with deep reflection eliciting deeper learning.
- Deep reflection is desirable for learners at all stages, from undergraduate students to experienced clinicians, as it allows exploration of the emotions associated with the learning experience, removing barriers to learning and supporting wellbeing.
- Further research to explore the barriers and facilitators to deep reflection in dentistry is indicated in order to support the planning and implementation of a systematic overhaul of current and often ineffective methods of reflection.

## Introduction

Reflection is an essential component of the learning process. It has been defined as 'the active, persistent and careful consideration of any belief, or supposed form of knowledge in the light of grounds that support it and the further conclusion to which it tends'.^1^ It is considered to be a deliberate and structured process requiring one to recapture and contemplate on real experiences and challenge existing beliefs.^2^^,^^3^

Reflection can help learners to bridge the gap between theory and practice, allowing them to find answers that they are unable to access through formal learning.^4^ In healthcare, reflection uses authentic, experiential activities to elicit a deeper form of learning, allowing the generation of 'transformative knowledge'; new knowledge that compels the clinician to change their practice behaviour.^5^^,^^6^

The practice of reflection is considered to offer broad and multi-faceted benefits. Deeper and more meaningful reflection has been associated with improved self-awareness, for holistic and lifelong learning.^2^ For dental students, reflection can help to develop professional identity and self-confidence, alongside challenging assumptions and stereotypes, improving communication skills and providing an enhanced awareness of the complexity of their patients' lives.^7^ Furthermore, it may help students to commit to the provision of service by providing quality care to make a difference, by gaining insight into the communities and lives of the patients that they care for.^7^ As such, reflection is essential for all practitioners of dentistry, from the undergraduate student to the hospital consultant or general dental practitioner.

A further benefit of reflection relates to wellbeing, a priority during the COVID-19 pandemic in particular. Learning in a clinical environment for healthcare professions such as dentistry can be especially stressful, causing negative impacts on students' physical and mental health.^8^^,^^9^Dental students may feel insecure regarding their contribution to patient care and their role within the dental team, which in turn, may present as a barrier to learning.^10^ Through exploring their feelings associated with a challenging learning experience, without external judgement, a student can be empowered to reinterpret these experiences in light of their inner strength and wisdom.^1^^,^^11^

Despite the well-evidenced advantages of reflective practice, it is clear that there are some inherent challenges in reflecting. Reflection itself is often not straightforward and requires underlying knowledge. Furthermore, it takes time and practice for the necessary skills to be developed, with Rolfe arguing that students can only learn to become reflective practitioners when they are in practice and supported to do so.^12^ Moreover, the ongoing need to evidence the value gained from reflection can be repetitive and lack meaning.

This paper aims to explore the existing literature on reflective practice in dentistry and identify areas for further research to improve reflective practice within dentistry. Key areas discussed will be: the requirement of reflection in dentistry; models of reflection that are relevant to dentistry; reflection through traditional means such as structured written reflections, logbooks and portfolios; reflective essays; and reflection with assessment. Furthermore, more novel methods of reflection including journals will be discussed, alongside barriers to reflection and recommendations for the future with respect to reflection in dentistry.

## Method

This is a narrative review of the literature for which the StarPlus database was searched. This is a University of Sheffield database which includes Google Scholar, PubMed, Medline, Ovid, Scopus, SAGE and ScienceDirect. The initial search on StarPlus was based upon the key words 'reflective practice', 'reflection', 'dental' and 'education'. This was expanded to include 'creative reflection' and 'healthcare' to explore more novel approaches that may not yet have been applied to dental education. Relevant legislative- and registration-related literature on reflective practice in dentistry was hand searched. The search was undertaken on 26 February 2020. The search results were reviewed by one author (FC) for relevance. The included studies were reviewed by both authors and a narrative review produced using the Scale for the Assessment of Narrative Review Articles (SANRA) criteria for quality of narrative reviews.^13^

This method has limitations in that some key literature may have been missed; however, this was intended to be an accessible and informative summary of the relevant literature, rather than a systematic review.

## Reflection as a requirement

Reflective practice is a key component of policy documents for providers of undergraduate dental courses, both in the UK and further afield.^14^^,^^15^^,^^16^ In the UK, this includes evidencing that reflective practice has been undertaken by students throughout their five years of training. The American Dental Association states in their Accreditation Standards for Dental Education Programmes that becoming a competent professional involves daily reflective practice.^16^ The benefits beyond undergraduate qualification have also been acknowledged by professional dental regulatory bodies globally, whereby it is understood that the development and maintenance of professional standards and skills involves rigorous self-assessment and reflection on one's current practice.^3^^,^^15^^,^^16^^,^^17^ In the UK, upon registration with the General Dental Council (GDC), registrants are required to undertake meaningful experiential learning on an ongoing basis and should be able to explain the importance of critical reflection.^15^^,^^18^

Furthermore, the GDC encourages qualified dentists to be reflective practitioners, whereby they should consider their experiences to gain insight into their practice to support the continual improvement of the quality of their care. In 2018, the GDC introduced the Enhanced Continuing Professional Development (ECPD) scheme, which requires all registrants to keep a record of relevant learning, including mandatory demonstration of reflection.^19^ The Australian Dental Board encourages continual reflection on current practice in directing one's continuing professional development (CPD), with some Australian boards detailing reflection as a requirement of the CPD.^17^^,^^20^ Reflection on the everyday clinical experience facilitates experiential learning and professional development and has been shown to have great value in complementing CPD for both individuals in recognised speciality training programmes and the experienced practitioner.^21^

## Models of reflection

A large range of reflective models have been proposed in the literature, varying in terms of the focus of the reflection (the person or the situation), the depth of the reflection (superficial or deep) and the perspective taken (individual or otherwise).^4^^,^^22^^,^^23^^,^^24^^,^^25^^,^^26^ It is beyond the scope of this paper to explore these models in detail. Nonetheless, one important model with significant relevance to dental education, described by Schön, proposes two aspects of reflection: reflection-in-action, which occurs during experience where one can respond by modifying behaviour immediately; and reflection-on-action, after the experience with consideration of the event with thought and feeling on this.^4^ Further to this, reflection-before-action has been described in nursing, where one reflects on what they want to do and how they intend to do it before they do it in order to avoid error and to provide an important opportunity for feedback.^27^

Rolfe identified the advantages of reflection-on-action in managing wicked problems: those unique and complex situations that one cannot prepare for in advance, whereby an individual must generate their own theory on how best to proceed and test it out in an on-the-spot experiment.^12^ In order to evaluate the outcome of the chosen approach, this reflective practice should be undertaken with others as a partnership or team.^4^^,^^12^ Rolfe acknowledges that while these types of challenges are not uncommon in any field of healthcare, the opportunity to reflect with others may be less available for some practitioners.^12^ The dental team is typically broad, comprising dentists, dental nurses, dental therapists, dental hygienists, dental technicians and administrative staff, though the majority of care is provided in much smaller units. The varied settings and teams within which dental care is provided may impact upon the opportunities an individual may have for reflective practice with colleagues.^12^ For example, a small team working within a dental practice may potentially have fewer opportunities for group reflective practice when compared to a large team within a dental hospital. Nonetheless, the coordination of time together for larger groups to reflect may be a challenge, when a small team may be able to organise more consistent, regular sessions for practice. Further research is necessary to explore this area.

## Reflective practice in dentistry

### Traditional methods of reflection

#### Structured written reflections

The literature regarding reflective practice in dental education has been limited, yet the overwhelming majority of this investigates the use of structured written reflections.^28^^,^^29^^,^^30^ One study explored the use of structured written reflections to assess the learning provided by a placement in conscious sedation for undergraduate dental students in the UK.^30^ The results suggested that this method engaged the students in reflecting on the challenges of the learning experience, with some providing strategies to overcome these in future.^30^ The students were given frameworks using both Rolfe and Gibbs cues, though only students whose structured written reflections were guided by Gibbs discussed their confidence. The frameworks can be seen in Table 1.^30^

**Table 1:** Examples of structures for written reflections that have been applied to research in undergraduate dental education based on Johns' Framework, Gibbs' Reflective Cycle and Rolfe's Model of Reflection^30^^,^^31^

**Example of structured reflection headings based on Johns' Framework31**	**Example of structured reflection headings based on Gibbs' Reflective Cycle30**	**Example of structured reflection headings based on Rolfe's Model of Reflection30**
What happened?	Describe the appointment	What? Description of event What did you see/do? what was your role? What did other people see/do? What was good/bad?
Describe your feelings at the time that this happened	What were you thinking and feeling when the appointment started?	So, what? Analysis of the event What did you learn? What could you have done better? What do you now understand? What were the effects of what you did?
Why do you consider this to be worthy of reflection?	What was good/bad about the appointment?	Now what? Proposed actions following the event Are changes required? What would you do next time? Consequences if you do not change? What information would you need for next time?
What strengths in your clinical practice did this experience demonstrate?	The procedure(s) that I performed/observed today helped my understanding of dental sedation because…	
What learning needs did this experience reveal to you?	How would do things differently (if at all) if you had a similar appointment?	
Which one learning need, disclosed by this experience, do you wish to address as a priority?	I feel/do not feel confident that I would be able to manage a similar situation when qualified because…	
Decide exactly what you would like to achieve in relation to your selected learning need, before completing 'target setting'	What further information/skills do you think you need?	

Examples of headings used in structured reflections in these studies are shown in Table 1. Similarly, structured written reflections undertaken by dental hygiene and therapy students in the UK through worksheets have been shown to evidence both superficial 'descriptive' and deeper reflection. This form of reflection alone can only hope to facilitate reflection-on-practice, as discussed earlier.^26^^,^^31^ Nonetheless, presently this remains the mainstay of reflective practice in many aspects of healthcare.^12^

#### Logbooks and portfolios

One common form of structured written reflection in dental education is the integration of reflection into a logbook or portfolio in which to record experiential learning and examples of reflective activity. Traditionally, logbooks are 'a collection of learning objectives and additional information concerning a specific educational period'.^32^ They are a record for the student and educator that help to structure clinical learning, with an overview of the requirements of training, including those outstanding and inform and include setting of learning plans.^32^ Meanwhile, a portfolio focuses on 'students' documentation and self-reflection of their learning activities'.

It is acknowledged that logbooks facilitate immediate and ongoing communication between learner and educator in the clinical environment, alongside providing a feedback loop for evaluating the learning activity and a method of continuous assessment.^33^ However, logbooks can often be inadequate, for reasons such as a learner perception of logbooks being boring, bureaucratic and an exercise in collecting signatures with no consequence for improper completion and a misalignment of clinical experience and logbook requirements.^32^^,^^34^Logbook completion is often compulsory, with reflections being completed alongside target setting.

Written forms of reflection such as logbooks have been combined with group discussion, mentorship and used as a facilitator for reflective discussions, with these additional elements being beneficial in promoting learning and facilitating reflection.^31^^,^^35^^,^^36^ These discussions and feedback between educators and learners which frequently occur in dental education during and after clinical sessions can be beneficial in stimulating internal reflection; however, this is not studied in the literature as a method of reflection. Furthermore, a qualitative study with undergraduate dental students undertaking clinical attachments in paediatric dentistry in the UK found that written reflection using logbooks alone may not facilitate reflection at all, due to barriers such as a perceived lack of understanding of and preparation for reflection and a greater emphasis placed by students and educators on learning through experience rather than reflection.^34^

Portfolios may also be used with undergraduate dental students to facilitate reflective practice.^37^ However, it has been acknowledged that students need support in learning to reflect in this manner.^37^ Assessment or review of these by staff is also labour and time intensive and students may have anxiety surrounding the opinion of those who can see this portfolio.^37^

Confidentiality concerns have been reported to affect the depth of reflection achieved by dental students in both the UK and Australia when using portfolios and written logbooks.^32^^,^^34^^,^^36^ Dental students in the UK reflecting through a written portfolio described that they modified reflections because they knew that their mentor was going to read it.^36^ Recent events in the UK have also threatened to undermine the safety of reflective practice in healthcare professions, whereby a reflective portfolio was utilised in a fitness to practise case for a junior doctor investigated for negligence in a manslaughter case.^38^ Although the reflections were not used in the criminal case proceedings, understandably this has led to heightened caution during reflective practice in healthcare, including dental education.

#### Reflective essays

There is plenty of evidence regarding the use of reflective essays on clinical experiences with medical students.^39^^,^^40^^,^^41^ They have been shown to be rewarding and to have enhanced the learning experience when medical students completed reflective essays on their experience during clinical placements in palliative care.^42^ These students, however, reported that the additional work of a reflective essay was burdensome, hoop jumping, contrived and intrusive.^42^ Furthermore, medical students in the UK and Australia have reported not reflecting honestly when required to do so through written essays and finding difficulty in expressing emotion.^42^^,^^43^

The term 'reflective essay' implies that reflection has taken place but it is uncertain whether it has and whether any reflection within the constraints of a formal essay is deep or meaningful. The reflective essay has often been used in medical education to assess student learning rather than to evidence reflective practice.^40^ It is interesting to note that the use of the reflective essay for dental students has not been studied in detail; however, in one study in the USA, undergraduate dental students and their educators did find value in reflective essays on critical incidents in facilitating reflection.^7^^,^^28^ Undergraduate dental education is frequently more focused on practical skills with greater and earlier clinical experiences than in medical education. This may invoke different emotions associated with the learning experience and thus require a different approach to reflective practice.

#### Reflection with assessment

Assessment of written reflections, such as essays, may cause students to modify what they write and give them a feeling that they were writing their reflection for someone other than themselves.^44^ Focus on assessment criteria can overtake that of reflecting deeply.^43^ Self-censorship of student reflection has been found to be more likely when students feel that what they are writing may be deemed by evaluators of the reflection as negative, which was demonstrated in the evaluation of reflection through worksheets by UK dental students.^31^^,^^45^This has led to debate on whether reflective writing should be assessed at all.^46^ The potential for reflective activities, such as poetry and storyboarding, to form the basis of assessments has also been explored with nursing and physiotherapy students and was demonstrated to be successful in facilitating reflection.^47^^,^^48^^,^^49^ Nonetheless, the compulsory nature of an assessment and the need for it to satisfy marking criteria may preclude the depth of reflection being undertaken by learners.

The impact of this may vary depending on whether the reflective piece is assessed formatively or summatively, but more research is needed to determine this. It has also been suggested that reflection-based assignments would be better suited for low-risk, formative types of assessment.^49^ A recent qualitative study exploring reflective practice for undergraduate dental students found enthusiasm from learners and educators in moving towards assessing engagement with the reflective process rather than assessment of the reflective practice itself, a move which would have to satisfy relevant regulatory bodies.^34^ Furthermore, the use of structured reflection to inform target setting, for example creating learning action points, has been shown to elicit an emphasis on negative aspects of the learning experience in dental therapy students.^31^

### Novel and creative methods of reflecting

There is a growing interest in the use of alternative and more creative modes of reflective practice in other fields.^50^ Application in nursing, for example, has suggested creative reflective practice, such as poetry, storyboards and artwork, may offer numerous advantages to structured written reflection, particularly for the development of emotional awareness surrounding issues such as death and serious illness.^47^^,^^51^^,^^52^^,^^53^ A particular advantage of these methods over traditional methods is the facilitation of the learner reflecting through a voice other than their own, allowing greater freedom to explore feelings.^53^ While these advantages were also observed with the use of a storyboarding reflective technique with student nurses, the researchers reported that this approach was both staff and time intensive, with particular attention necessary to develop a climate of trust and safety.^53^ Outside of healthcare, poetry has been shown to support reflective practice in a group of student teachers, particularly in reflecting on their motivation for choosing that profession.^54^ Similarly, creative approaches have been employed with sports coaching students, where they were found to be useful in facilitating reflection on prior learning and the formation of action plans.^48^

#### Journals

The integration of journals, also referred to in the literature as reflective diaries, into undergraduate dental courses has been reported to assist in formalising reflection, providing an outlet for personal feelings and an opportunity for feedback about students' experience of the course and a means to provide insight for both students and educators into the learning process.^35^^,^^55^ There are, however, issues with the application of reflective journals in dentistry with respect to confidentiality and assessment, thus careful consideration must be given to fostering a trusting environment for reflecting through journals and appropriate education in their use.^35^

In dentistry, the literature has indicated that the processes of 'blogging' or keeping a clinical journal may be useful reflective learning tools, yet there is currently little evidence to support the effectiveness of these approaches or the use of more creative methods in this field.^29^^,^^56^ One study with dental students in the USA encouraged methods including the use of photography of the clinical setting but not the procedure or clinical experience directly, alongside discussion of the images captured and small group reflective discussions, in addition to more traditional methods of reflection.^28^ Students reported that it made their clinical experience more meaningful, interesting and rewarding.^28^ When blogging was used with dental hygiene students in the USA, quantitative analysis found an improvement in the depth of reflection reached by students practising blogging, in turn developing critical thinking skills, assessed through the California Critical Thinking Skills Test.^56^ However, this study had a small sample size, with only 11% of students participating in the study completing the blogs.^56^

The use of short video vignettes as a reflective exercise for undergraduate dental students in the UK was successful in facilitating reflection for both individuals when they were creating the video and for peers when viewing the video together.^57^The learning experience, including that of holistic care, was enhanced and reinforced and students learnt about the benefits of reflecting on more negative experiences, with enhanced depth of reflection.^57^ This was viewed positively by student participants; however, it was acknowledged that this approach will not suit everyone, such as more introverted students, with some expressing feeling camera shy and nervous of sharing their feelings.^57^ Despite a growing body of evidence to support the use of creative approaches to reflection in other fields of healthcare and education, there is a need for further research to determine the applicability, relevance and success of these techniques in dental education.

### Barriers associated with all methods

There are barriers to reflective practice in its various forms that may preclude some individuals from engaging.Further to the aforementioned limitation in opportunities for those working in small teams to reflect with others, a key barrier to reflection is the requirement for protected, dedicated time to undertake reflective practice.^3^^,^^32^ The literature suggests that the setting and time available for reflection can affect the depth of reflection undertaken.^5^ Moreover, the learner must be motivated to reflect, acknowledging that the process of reflection itself may produce an 'inner sense of discomfort', which may deter them from proceeding.^58^ It has also been suggested that learners may avoid reflective queries that require additional effort to process or access the necessary information.^31^ Most importantly, the ability to reflect is not innate; it is considered to be a skill that should be both taught and practised, a perception echoed in groups of students and educators in dentistry.^5^^,^^32^

Students have reported not knowing how to reflect, with a lack of formal teaching on this, contributing to knowledge being a perceived barrier to reflective practice for both student learners and educators in a qualitative exploration of reflective practice among undergraduate dental students.^34^ Improved and formalised teaching on reflective practice to ensure learners and educators have a good understanding of what reflection is and its importance may combat the instinct to focus reflection on negative learning experiences. Despite the emphasis on reflective practice from all stakeholders involved in dental education, there is a lack of clarity on how best to practically implement the facilitation of effective methods of deep reflection.

It has been frequently reported that students do not appreciate the value of reflection, nor enjoy reflecting,^31^^,^^34^ with 'the language of reflection being alien to most students'.^59^ However, it must be noted that the process does not need to be enjoyable for it to be valuable to students, yet lack of enjoyment is likely to be a barrier to completing an 'onerous' activity.^31^ Furthermore, when solely reflecting on a clinical scenario, students often focus on negative emotions and perceived weaknesses.^60^

Reflection is a compulsory aspect of dental education in the UK.^14^^,^^15^ When reflection is mandatory, undergraduate dental students have reported feelings of resentment towards it.^31^^,^^35^ The requirement for individuals to be open and honest may provoke a strategic and sometimes hostile response to reflection, which has led to questions on whether reflective practice should be a compulsory exercise at all.^61^

## Recommendations for the future

The traditional methods of reflecting in dentistry discussed in this paper are so ingrained in students, educators and practising dentists, so heavily utilised by education providers and so extensively relied upon by regulatory bodies, that a systematic overhaul would be required in order to introduce more effective means of facilitating deep reflection. It has been suggested that reflection can be effectively learnt when students feel that they are in a safe and caring environment and are not at risk of being penalised.^62^ Thus, creating a safe space for reflection that is not assessed is necessary.

This overhaul would involve a shift away from structured written reflections, more research into creative approaches of reflection to demonstrate the ability to facilitate deep reflection in dental education and the exploration of alternatives to compulsory assessed reflective practice. This may include greater student autonomy on how and when they reflect, with varied opportunities to reflect being offered. While the GDC doesn't specify how practising dentists should reflect, or evidence this within ECPD in the UK, it must be established whether regulatory bodies would approve of these alternative methods of reflection being integrated into the undergraduate dental curriculum; more specifically, assessing student engagement with the reflective process, rather than the content of the reflection.

## Conclusion

In conclusion, reflective practice has a clear role throughout dental education, from undergraduate level to ongoing registration with regulatory bodies. The benefits of reflective practice identified from educational research and studies in other areas of healthcare may be easily relatable to dentistry, though there is little evidence to inform the most appropriate format for reflective practice in dental education. There is a need for further research to determine the effectiveness of reflective practice in dental education where there is arguably less available evidence, particularly as a move away from structured written reflection to more creative reflective opportunities are encouraged. Dental and dental hygiene and therapy students are unique within healthcare education, owing to their clinical exposure, experience and responsibility in providing operative treatment to their own patients at an early stage in their undergraduate training. Furthermore, dentists and dental care professionals encounter different challenges in their practice. Unlike medical or nursing students, most dental students will immediately assume a management and leadership role in their team within a dental surgery. Moreover, greater exploration of the barriers to reflection in dentistry is indicated, with consideration to how these may be overcome and a need to engage regulatory bodies in system-wide changes.
